# Cytoscape Automation: empowering workflow-based network analysis

**DOI:** 10.1186/s13059-019-1758-4

**Published:** 2019-09-02

**Authors:** David Otasek, John H. Morris, Jorge Bouças, Alexander R. Pico, Barry Demchak

**Affiliations:** 1 Department of Medicine, University of California, La Jolla, San Diego, CA 92093 USA; 2 University of California, San Francisco, San Francisco, CA 94143 USA; 3 Bioinformatics Core Facility, Max Planck Institute for Biology of Ageing, Cologne, Germany; 4 Gladstone Institutes, San Francisco, CA 94158 USA

**Keywords:** Workflow, Reproducibility, Cytoscape, Interoperability, REST, Microservice, Service-oriented architecture

## Abstract

Cytoscape is one of the most successful network biology analysis and visualization tools, but because of its interactive nature, its role in creating reproducible, scalable, and novel workflows has been limited. We describe Cytoscape Automation (CA), which marries Cytoscape to highly productive workflow systems, for example, Python/R in Jupyter/RStudio. We expose over 270 Cytoscape core functions and 34 Cytoscape apps as REST-callable functions with standardized JSON interfaces backed by Swagger documentation. Independent projects to create and publish Python/R native CA interface libraries have reached an advanced stage, and a number of automation workflows are already published.

**Electronic supplementary material:**

The online version of this article (10.1186/s13059-019-1758-4) contains supplementary material, which is available to authorized users.

## Introduction

As a platform for network biologic analysis, Cytoscape [1] has proven to be enormously popular, with over 17,600 downloads worldwide each month, 5000 startups each day, and over 1000 direct citations per year. Investigators can interactively explore complex *omics datasets via analysis and visualization functions provided by Cytoscape and a large and vibrant community of app contributors. However, interactive use has proven inadequate for precisely reproducing or sharing complex analyses or for scaling to high volume or production analysis. Moreover, while Cytoscape apps provide highly performant and relevant network biology functionality, the specialized programming talent and relatively long development times they require can make them uneconomical for delivering complex and evolving workflows. Finally, as an interactive tool, Cytoscape is not positioned to add value to emerging workflows that integrate one or more external data acquisition and analysis tools (e.g., Galaxy [2], Taverna [3], and libraries provided in repositories such as PyPI [4] and Bioconductor [5]).

**Fig. 1:**
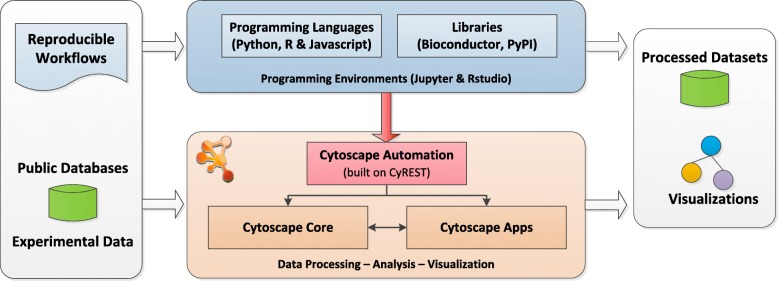
Overview of the Cytoscape Automation ecosystem. Reproducible workflows (as Python/R/Javascript or Cytoscape Command scripts) and datasets control Cytoscape through Cytoscape Automation. Results can be created either directly from Cytoscape or from Python/R/Javascript themselves

As shown in Fig. 1, Cytoscape Automation [6] is a new Cytoscape feature that addresses these issues by extending the existing CyREST [7, 8] app, which empowers bioinformaticians to create reproducible workflows expressed in robust and well-known programming languages (e.g., Python, R, Javascript) using familiar programming environments (e.g., Jupyter and RStudio). Under Cytoscape Automation, workflows can use CyREST to issue commands to Cytoscape and automation-enabled apps via the REST protocol, which encodes data as JSON documents. Both REST and JSON are already in wide use in client/server computing, are accessible from most programming languages, are immediately understood by most bioinformaticians, and are easy to learn given the massive body of relevant training materials, examples, and extant community.

This paper focuses on using Cytoscape Automation from Python and R because they are widely used and understood by bioinformaticists and because they already have well-documented repositories of bioinformatic functions that enable researchers to create reliable, flexible, and performant bioinformatic workflows quickly and easily. Our py2cytoscape [9] (for Python) and RCy3 [10] (for R) libraries provide easy access to Cytoscape and app functionality and are available in these repositories, too. Library download statistics reported by GitHub, PyPI, and Bioconductor indicate that researcher interest in Cytoscape Automation is strong—500 downloads/month for py2Cytoscape and 800 downloads/month for RCy3.

Critically, Cytoscape Automation creates new standards that encourage Cytoscape core and app authors to expose Cytoscape functvionality via REST-based API calls backed by state-of-the-art documentation based on the widely used Swagger [11] documentation framework. Swagger is purpose-built to improve workflow author productivity in a REST context by presenting complete CyREST endpoint documentation, organizing endpoints by category, and assisting in workflow prototyping via an easy click-to-run web-based interface.

As a result, novel network biologic workflows can now be quickly and cheaply delivered as integrations of Cytoscape functions, complex custom analyses, and best-of-breed external tools and language-specific libraries.

In this paper, we explain the key features of Cytoscape Automation, including how they work, how Cytoscape app developers can make automation-enabled apps, and how workflow authors can leverage Cytoscape Automation to create and evolve their workflows. As a reference material, we provide the substantial Cytoscape Automatic Wiki [12], which contains articles on context, implementation details, FAQs, best practices, and sample scripts and apps to help workflow authors become quickly productive and help Cytoscape app authors produce new automation-capable apps or upgrade existing ones.

As an illustration aid, we use the running example of Cytoscape Diffusion [13], which uses network propagation to find new nodes (e.g., genes) that are most relevant to a set of well-understood nodes. Diffusion is particularly apt because it shows how to define real-world CyREST endpoints that are well documented, consume network and customization parameters, and produce actionable results.

In this paper, the “Design” section describes the components of Cytoscape Automation and explains their use. The “Implementation” section outlines the details of CyREST construction, and the “Results” section presents concrete examples of Cytoscape Automation benefits. The “Discussion” section compares Cytoscape Automation to other biological workflow environments. Finally, the “Future development” section calls for additional contributions that can expand Cytoscape Automation to improve workflow economics even further.

Note that this paper describes CyREST v3.8, which is included with Cytoscape v3.7.0 as a core app, meaning that it is automatically synchronized with Cytoscape by the Cytoscape developer team. Cytoscape Automation requires CyREST v3.8, and we highly recommend that users running Cytoscape versions earlier than v3.7.0 upgrade to v3.7.0. As CyREST evolves, it will be disseminated in new Cytoscape releases and via the Cytoscape App store. CyREST follows semantic versioning guidelines [14], thereby guaranteeing that updates will not break the workflows or automation-enabled apps as it evolves. We highly recommend that independent app developers conform their apps’ evolution to semantic versioning principles, too.

## Design

The leap from the original CyREST implementation to address the broader scope of the Cytoscape Automation initiative required new features and upgraded approaches in a number of technical areas:

❶ New CyREST access to Cytoscape apps
❷ New CyREST access to Cytoscape Command script operations
❸ Improved documentation infrastructure and content standards
❹ New interactive CyREST call prototyping
❺ Consistent mechanisms for calling CyREST and receiving return values
❻ Improved coverage of core Cytoscape functionality

**Fig. 2:**
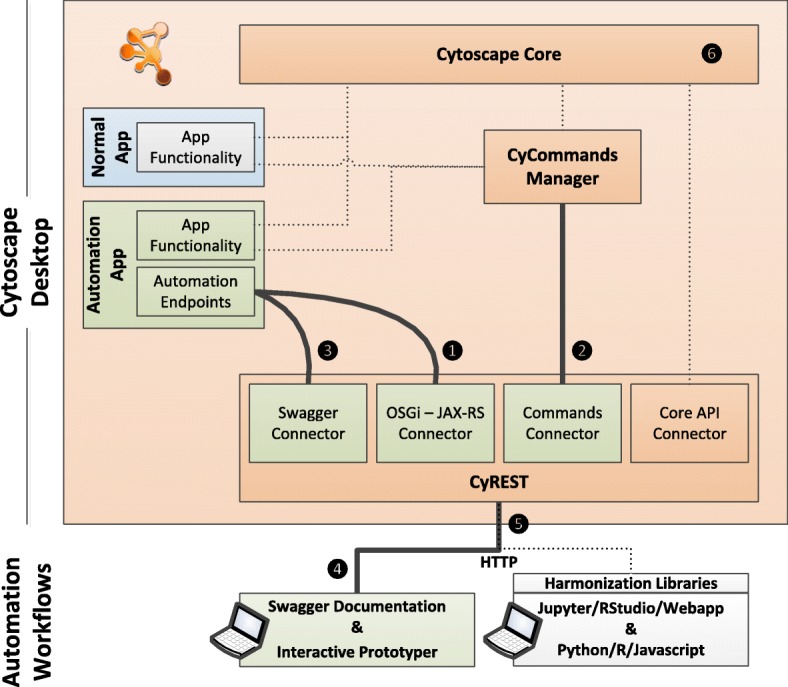
Relationship between the Cytoscape Desktop (including CyREST, Cytoscape apps and Cytoscape core) and Cytoscape Automation workflows. Dotted lines indicate command/data flows that pre-date Cytoscape Automation. Solid lines indicate flows created for Cytoscape Automation. New components are in green

Figure 2 shows the relationship between the Cytoscape Desktop and Cytoscape Automation workflows. The Cytoscape Desktop includes both the Cytoscape core (including CyCommands and CyREST) and apps sourced from the Cytoscape App store. Automation workflows execute outside of Cytoscape but use CyREST to leverage Cytoscape features. Figure 2 is annotated to show the components important in each facet of the Cytoscape Automation design, which are described in this section.

Note that for a workflow to access Cytoscape functionality, Cytoscape must be running and accessible via HTTP calls from the workflow execution environment.

Note that calling a CyREST endpoint requires the use of REST interface functions found in most modern languages. While all CyREST endpoints are accessible in this manner, we have created harmonization libraries for R and Python (described in the “Implementation” section) to enable quick and easy access to common Cytoscape Automation features. However, direct CyREST calls are required for all other endpoints, including those supplied by Cytoscape apps—see the “Python and R Harmonization Libraries” section for details.

### New CyREST access to Cytoscape apps ❶

A large part of Cytoscape’s utility to researchers is provided by apps—their inclusion in Cytoscape Automation, facilitated by CyREST, greatly expands the functionality that can be leveraged via scripting workflows. Apps that support automation (called Automation Apps) can expose functionality either via a Function or Command interface [15].

A Function interface enables a script to pass complex parameters and receive return results of arbitrary length and complexity. While Functions can be called from scripting languages such as Python, R, and Javascript, they cannot be called from the Cytoscape Command Tool [16].

To create a Function in an existing app, the app author must add a new function that defines a CyREST endpoint using JAX-RS [17] annotations and which executes app-related code—most likely code that implements existing app functionality. The JAX-RS annotations define the endpoint name, the HTTP protocol [18], and the parameters to be passed. For example:

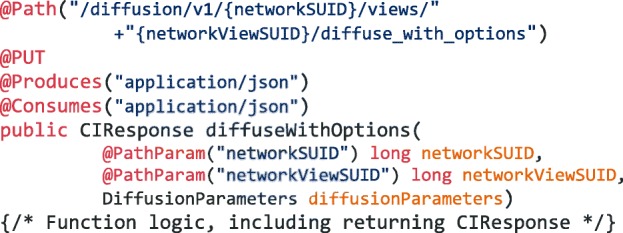



This defines the diffuse_with_options endpoint that accepts three parameters (networkSUID, networkViewSUID, and diffusionParameters) and returns a CIResponse structure. The @PUT annotation defines the HTTP method (as PUT), and the @Path annotation defines the endpoint path (/diffusion/v1/{networkSUID}/views/{networkViewSUID}/diffuse_with_options), which the client appends to CyREST’s base URL (http://localhost:1234) when calling diffuse_with_options. The @Produces and @Consumes annotations define the PUT payload and response as JSON [19].

An actual CyREST URL that calls diffuse_with_options might appear as http://localhost:1234/diffusion/v1/53/views/744/diffuse_with_options and would include JSON corresponding to the DiffusionParameters class as the HTTP PUT payload. For diffuse_with_options, a sample DiffusionParameters payload is:

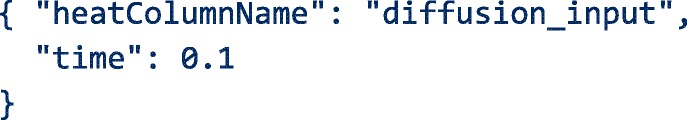



As shown in Fig. 2, at runtime, CyREST’s JAX-RS connector parses the URL to extract the networkSUID and networkViewSUID values and parses the PUT payload to create a DiffusionParameters instance. JAX-RS calls the diffuse_with_options function with these values, which performs a diffusion operation and returns a CIResponse instance. Finally, JAX-RS encodes the CIResponse into JSON and returns it to the caller.

The process for exposing app features as Commands is different, as explained below.

### New CyREST access to Cytoscape Command script operations ❷

A Command interface enables a script to execute Cytoscape Commands analogous to commands executed within a Unix or Windows terminal, and they offer similar argument structure and execution. Command executions can pass simple parameters and can return results of predefined length.

Users can execute Commands as single lines (via Cytoscape’s Command Tool [16]) or as scripts (either via Cytoscape **Tools** → **Execute Command File** menu or on the Cytoscape command line via the -S parameter). Scripting languages such as Python, R, and Javascript can execute them via CyREST using an HTTP POST operation and passing Command parameters as a JSON object. The endpoint path begins with /v1/commands and is followed by the Command namespace and the command name. A fully formed URL and POST payload for the diffuse_advanced Command is:

http://localhost:1234/v1/commands/diffusion/diffuse_advanced

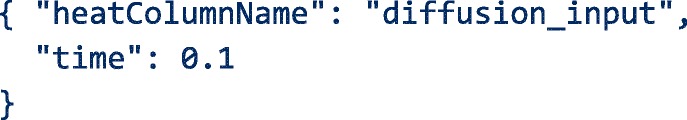



As shown in Fig. 2, the Commands system leverages the Cytoscape Tunable/Task system [20] (i.e., CyCommands Manager and Cytoscape Core) originally defined to collect execution parameters via a dialog box and then execute a Java function. The function consumes the parameters, performs the Command operation, and possibly returns a result as a fetchable task state.

To create a Command in an existing app, the app author must first register the Command’s namespace and name in a TaskFactory via the app’s CyActivator. The name must be the name of a public function within the app, and the app author must add @Tunable annotations to the function to define Command parameters.

If an app already exposes a function as a Tunable/Task, enabling the function to be called as a Command can be as simple as registering the TaskFactory with a suitable namespace and name. If an app does not use Tunable/Tasks, it may be easier to expose app features as Functions (as described above).

Note that in CyREST previous to Cytoscape v3.6, Commands were available via an HTTP GET operation, where parameters passed on the URL (e.g., ?time = 0.1) and the result form and content was not JSON, and they varied with the Command. The GET form has been deprecated in favor of POST to allow more parameters and to enable JSON-structured parameters and return results.

### Improved documentation infrastructure and content standards ❸

In the process of implementing Python and R support libraries and providing support for researchers producing scripting workflows, we found that the coverage and quality of CyREST’s Miredot-based [21] API documentation was a major impediment to productivity. We replaced Miredot with the popular Swagger [11] framework, which organizes CyREST endpoints by category, provides for more complete documentation, and presents an easy click-to-run web interface. This allowed us to more rigorously define and enforce the documentation standards that define an endpoint contract, including the context, purpose, caveats, parameters, and return results for each endpoint. Swagger also facilitates the documentation of structures (called *models*) pertinent to parameters and return results.

To access Swagger for Functions, use Cytoscape’s **Help → Automation → CyREST API** menu. For Commands, use **Help → Automation → CyREST Command API**.

For each Function and Command implemented in Cytoscape Core, we audited the documentation to verify that it contained meaningful and actionable content for each Swagger section according to best practices. Similarly, Automation App authors wrote their Swagger page documentation to the same standards.

For Functions, the CyREST Swagger Connector (see Fig. 2) synthesizes an endpoint’s Swagger documentation from text embedded in annotations attached to endpoint code. For Functions, a basic contract is defined by the @ApiOperation and @ApiParam annotations, which describe the endpoint generally and its parameters specifically. For the diffuse_with_options Function, these annotations might appear as follows:

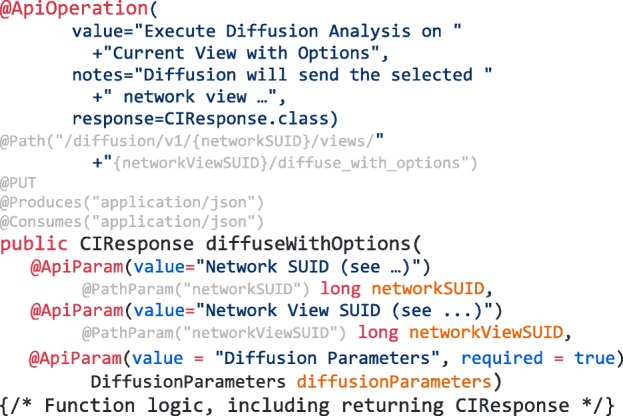



In the @ApiOperation annotation, the value attribute contains a short description; the notes attribute contains the context, purpose, and caveats, and the response attribute identifies the model (i.e., class) for the return result. The @ApiParam annotation applies to each parameter, whether it is part of the URL (e.g., networkSUID and networkViewSUID) or the PUT payload (e.g., diffusionParameters). The value attribute describes the parameter, while the required attribute indicates whether the parameter must be present. Additional annotations describe possible results and models.

**Fig. 3:**
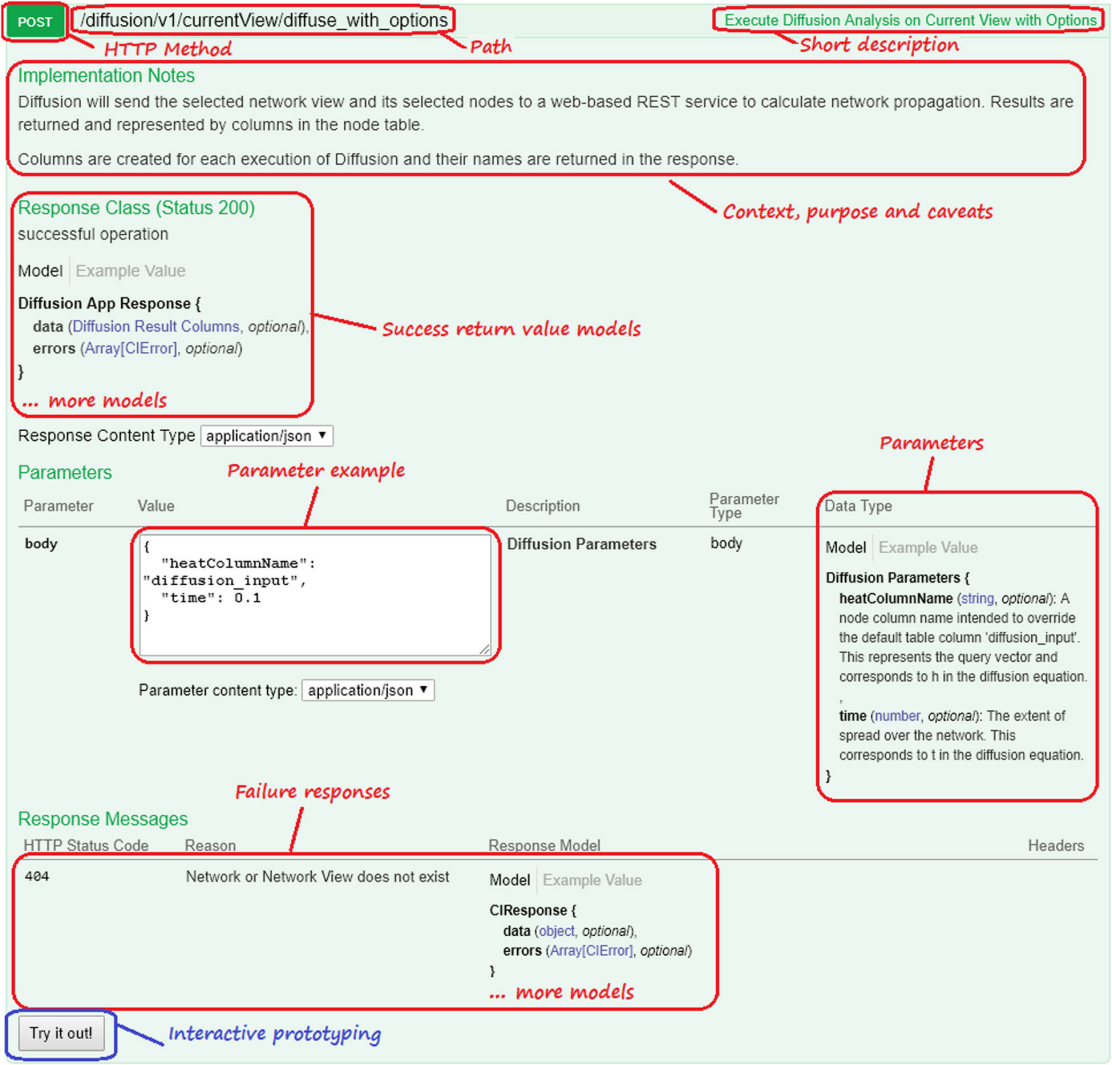
Sample Swagger page for diffuse_with_options, including markups for key areas. The *Try it out!* button calls Cytoscape to execute this CyREST function

Figure 3 shows a sample Swagger page corresponding to the diffusion_with_options Function above.

For Commands, the CyREST Swagger and Commands Connectors (see Fig. 2) synthesize a similar page from OSGi properties and annotated fields within a TaskFactory. Command-level descriptions, for example, are synthesized from attributes supplied in the TaskFactory properties when the task is created in CyActivator:

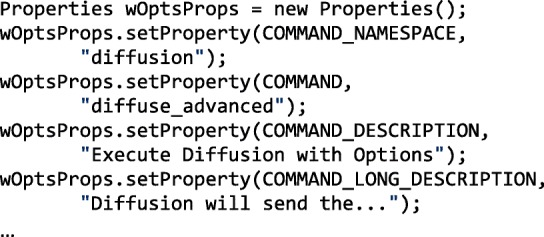



Parameter-level descriptions are synthesized using @Tunable annotations applied to variables within each TaskFactory:

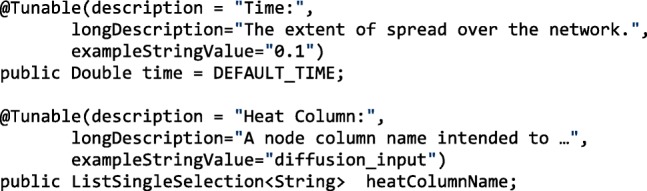



### New interactive CyREST call prototyping ❹

A significant cost in most workflow authoring processes is experimentation with library functions to determine what types of calls achieve workflow goals. The Swagger documentation system addresses this in an innovative way by enabling a user to formulate and submit a CyREST endpoint call directly from the endpoint’s Swagger page.

**Fig. 4:**
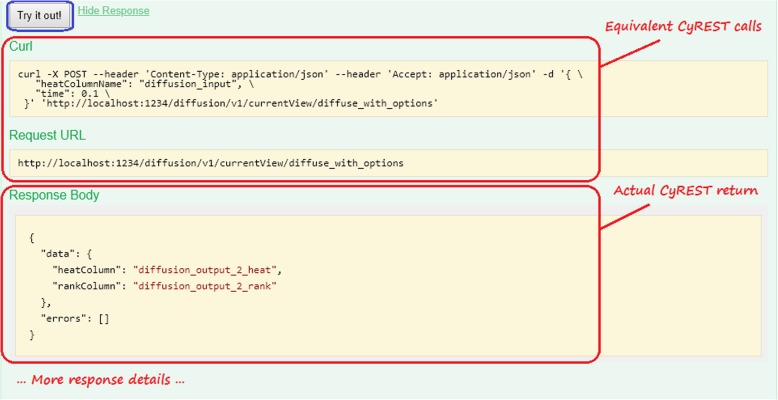
Sample Swagger results from using the *Try it out!* button to execute a CyREST call. The page shows the CyREST call that incorporates user-specified parameter values and the JSON-formatted call results

Using the example in Fig. 3, once the user fills the endpoint’s parameter values, provided by the included Example Value, clicking on the *Try it out!* button results in a well-formed diffuse_with_options call to Cytoscape, which performs a diffusion and returns a result (as shown in Fig. 4). If the diffusion fails, an error result is returned. By experimentation, and without *any* programming skills, a user can quickly understand and productively use a CyREST endpoint, which informs the correct composition of a REST call using the workflow language’s REST interface.

Note that parameters for some endpoints are references to Cytoscape objects represented by SUIDs (e.g., POST /diffusion/v1/{networkSUID}/views/{networkViewSUID}/diffuse_with_options). A user can discover Cytoscape SUIDs by using Swagger to execute query endpoints (e.g., GET /v1/networks/currentNetwork).

### Consistent mechanisms for calling CyREST and receiving return values ❺

To improve workflow author productivity, we created conventions for the data returned by CyREST endpoints and we revitalized the Python and R harmonization libraries (called py2cytoscape and RCy3).

But for minor exceptions, all CyREST endpoints now return their results in a standard JSON data structure called CIResponse [22], which has two main elements: data and errors. If the endpoint is successful, the endpoint returns its result in data and leaves errors empty—the exact result is endpoint-dependent and is described in the endpoint’s Swagger page. For example:

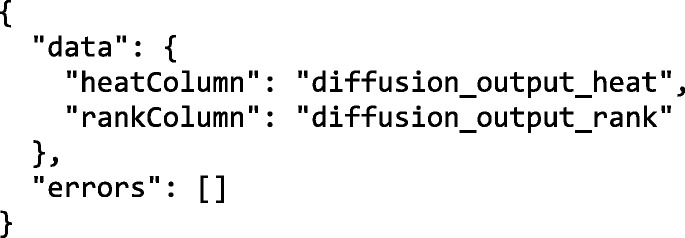



If the endpoint fails, it leaves data empty and returns errors, where errors[0] describes the endpoint error, and subsequent errors entries describe failures in any nested services that caused the endpoint failure, similar to a Java stack trace. For example:

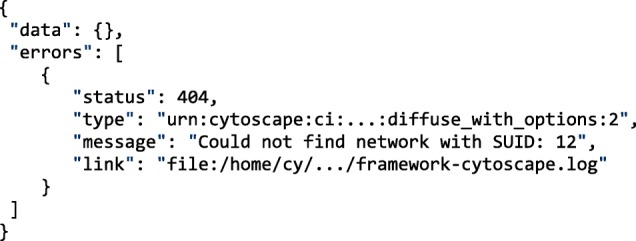



The status contains an HTTP status describing the error. Type contains a URN unique to the endpoint and error (shortened here for readability, but actually containing “urn:cytoscape:ci:diffusion-app:v1:diffuse_with_options:2”), and the message describes the error in prose. If the caller needs to take action for one type of error as compared to another, it should compare the type value, not the message content. The link value is returned but is not used.

The separation of data and errors enables callers to centralize their CyREST calling code, thereby easing coding and maintenance burden on workflow and harmonization library authors. A centralized CyREST caller should return the data value and throw an exception if an error is received.

### Improved coverage of core Cytoscape functionality ❻

Under Cytoscape Automation, the exposure of Cytoscape’s API via CyREST expanded from 113 available operations to 157. These new operations, as well as the data they consume and produce, are consistent with previous implementations. This API consistency follows the same Semantic Versioning [23] best practices laid out for Cytoscape core development.

## Implementation

The technical foundation of Cytoscape Automation is CyREST, which was first implemented by [7]. While Cytoscape Automation includes CyREST, it also includes harmonization libraries that enable Python and R workflows to easily make CyREST calls. In this section, we describe the implementation of all of these components. While Swagger is integral to Cytoscape Automation, too, it is a separate (free) product maintained separately.

### Defining endpoints

Originally, CyREST used an embedded Grizzly HTTP server [24] to publish Java resources annotated using the Jersey JAX-RS library [25] as REST endpoints. CyREST continues to support app Functions defined by JAX-RS-based endpoint annotations, though the Grizzly server and Jersey library were replaced by the OSGi JAX-RS Connector library (see Fig. 2) combined with the Jetty server contributed by Cytoscape’s Karaf [26] component. This connector library listens for services registered within the OSGi environment (e.g., Cytoscape Automation-enabled apps), recognizes any that have been provided with JAX-RS annotations, and then processes them the same way as CyREST’s internal JAX-RS resources.

By definition, Cytoscape apps have the capability to register services within Cytoscape’s OSGi environment. As described in the “Design” section above, adding REST endpoints is a matter of creating JAX-RS-annotated classes and methods and registering them as services. When the app registers services with OSGi, the OSGi JAX-RS Connector library recognizes the annotated endpoints and adds them to its Cytoscape Automation repository as Functions.

Cytoscape apps are also capable of registering Cytoscape Commands. A Cytoscape Command is an implementation of the Cytoscape TaskFactory interface which is registered as a service with OSGi. Any command added to Cytoscape’s CyCommand Manager is available to the Commands Connector, which consumes HTTP parameters, and passes them to the CyCommand Manager to perform the Command operation.

### Interfacing to Swagger

As described above, an app author should provide Swagger annotations to define the Swagger documentation for app Functions (as described in the “Design” section above). The Swagger Connector (see Fig. 2) harvests these annotations when the user requests via Cytoscape’s **Help → Automation → CyREST API** menu and then composes a Swagger-defined JSON object that represents both the endpoint category list and the endpoint documentation pages themselves. To display the app’s functions in a Swagger page, Cytoscape launches a browser to load the Swagger UI (hosted by Cytoscape itself as http://localhost:1234/v1/swaggerUI/swagger-ui/index.html), providing the JSON object as a parameter (as the http://localhost:1234/v1/swagger.json URL).

A parallel mechanism offers Swagger documentation for Commands, accessible via Cytoscape’s **Help → Automation → CyREST Command API** menu. App authors should provide OSGi properties and TaskFactory-annotated fields to define the Swagger documentation for app Commands. The CyCommands Manager provides this documentation to the Commands Connector, which translates that documentation to Swagger-defined JSON. To display the app’s Commands in a Swagger page, Cytoscape launches a browser to load the Swagger UI mentioned, this time providing the command JSON as a parameter (as the http://localhost:1234/v1/commands/swagger.json URL).

Note that Swagger document shows pages for all endpoints that were defined when the JSON object was retrieved by the Swagger UI. If the user installs or uninstalls additional apps, the user can refresh the browser window to re-fetch and view the corresponding updated Swagger JSON object.

Note, too, that the Swagger JSON object is available to any application that would like to enumerate the endpoints exposed by CyREST.

### Upward compatibility with previous CyREST

While Cytoscape Automation incorporates CyREST endpoint conventions described above, endpoints supplied by previous CyREST versions did not. Particularly, they did not return results in the CIResponse structure (described in the “Design” section).

The older CyREST Function endpoints returned a variety of JSON. To provide better and more uniform service, CyREST now offers the option of wrapping these endpoints’ return values in a CIResponse structure if the caller sets the CIWrapping: true HTTP header in the REST call. For example, the old-style response for GET http://localhost:1234/v1/networks.names is shown in green, and the CIResponse wrapper is shown in red:

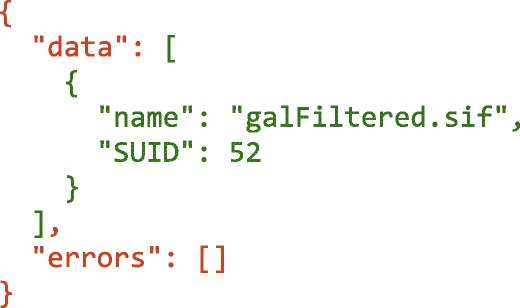



Also, all Command endpoints previously used the HTTP GET method, which relies on endpoint parameters being supplied as part of the REST URL. (Current conventions call for using the POST/PUT methods, which allow parameters to be expressed as JSON and passed as the HTTP payload.) The GET-based Command endpoints returned unformatted plaintext and could not effectively convey the details of any errors encountered.

CyREST continues to support the original GET Command endpoints, and any data they return, though the GET endpoints are deprecated. For every Command, a POST method using JSON parameters and JSON return (wrapped in a CIResponse object) has been added. The default Command Swagger references these POST methods exclusively to encourage their use while CyREST still supports the deprecated GET implementation.

### Calling endpoints

Cytoscape Automation simplifies Python- and R-based access to CyREST endpoints via harmonization libraries separately created, documented, and maintained by the Cytoscape community. The harmonization libraries provide language-specific and language-appropriate access to Cytoscape by wrapping one or more CyREST endpoints. As we add more CyREST endpoints, we believe the Cytoscape community will add functionality to take advantage of them. (Until new functionality is added to the libraries, direct CyREST calls via language-specific REST libraries remain necessary, as described below.)

The Python library (called py2cytoscape [27]) is available via PyPI. The lead developer is Jorge Boucas.

The R library (called RCy3 [28]) is available via BioConductor. The lead developer is Alexander Pico.

CyREST endpoints not covered by harmonization libraries can be called directly using REST protocols documented via Swagger. Endpoints contributed by installable apps (e.g., aMatReader) can either be called directly or, if implementing Commands (e.g., Diffusion), by generalized functions included in the harmonization libraries.

For example, a call to an aMatReader app function in Python would be made directly using CyREST, while a call to a Diffusion app function could be made either directly or via py2cytoscape:

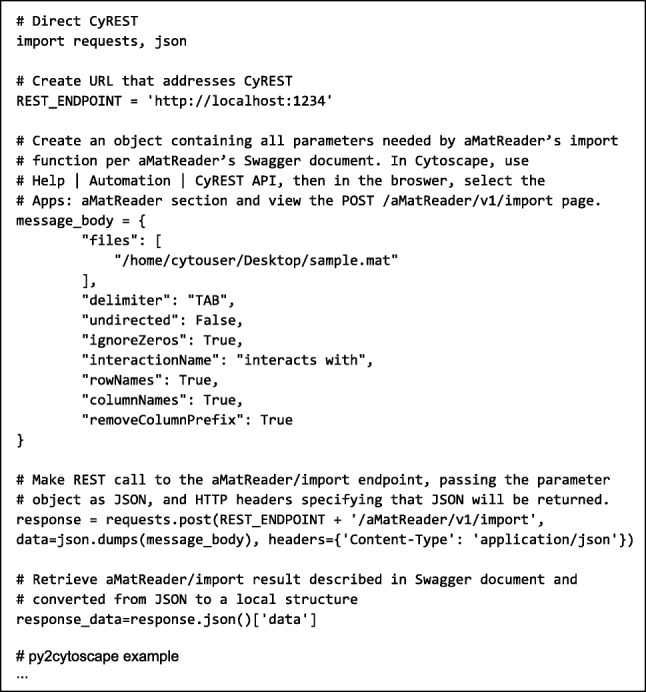



A call to the same aMatReader app function in R would be made directly using CyREST, while a call to a Diffusion app function could be made either directly or via RCy3:

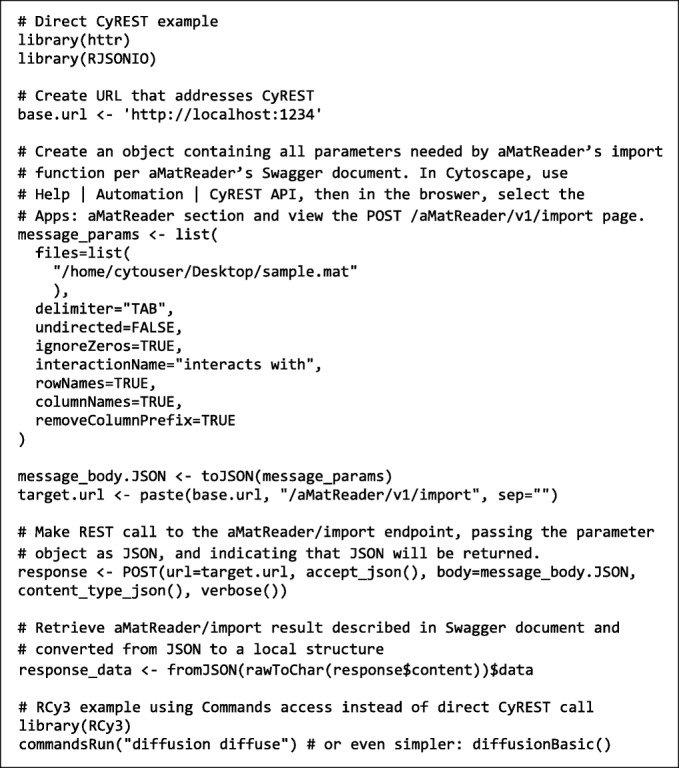



For apps that implement Commands, we provide a standard way to call their functions without necessitating the app-specific harmonization libraries, thus diminishing the need for direct CyREST calls. We also encourage app authors or community members to create and disseminate customized or extended app-specific libraries as well.

### Workflow examples

The Cytoscape community has created and published a number of sample workflows [29] based on both the Python and R harmonization libraries.

The following are the Python examples [30]:

*Advanced cancer networks and data*—retrieve disease networks from a public database and apply gene expression and tumor mutation datasets for network analysis and visualization. Network files and images are generated in multiple formats for sharing and publishing.

*Network Assisted Genomic Analysis (NAGA)*—re-prioritizes significant single nucleotide polymorphisms (SNPs) to genes using network diffusion methods including random walk and heat diffusion.

*Advanced View API*—demonstrates how users can manipulate Cytoscape network views directly from Python code.

The following are the R/notebook examples [31]:

*Cytoscape Automation with RCy3*—three use cases are demonstrated including querying existing interaction databases with a set of genes to create a network, creating a correlation network using aMatReader, and a basic enrichment analysis.

*Cancer networks and data*—retrieve disease networks from a public database and apply gene expression and tumor mutation datasets for network analysis and visualization. Network files and images are generated in multiple formats for sharing and publishing.

*AP-MS network analysis*—describes how to use data from an affinity purification-mass spectrometry experiment to generate relevant interaction networks, enriching the networks with information from public resources, analyzing the networks, and creating effective visualizations.

The following are the examples from the 2017 CyREST Challenge [32]:

*Konig_SBML_Time_Course_Python*—Python-based dynamic visualization of SBML kinetic models in Cytoscape.

*Grimes_CFN_CCCN_R*—R-based visualization of a cluster-filtered network (CFN) and a co-cluster correlation network (CCCN).

*Isserlin_PPI_network_pipeline_R*—R-based visualization of all interactions in a ligand-receptor network.

In the future, we hope to provide a standard way for workflow authors to create and disseminate workflows they create.

## Results

Cytoscape Automation was first released as part of Cytoscape v3.6 on November 15, 2017, and has been downloaded and installed over 300,000 times since then. In that period, Cytoscape was started over 550,000 times. Additionally, demand for our Cytoscape Automation workshops has been brisk. To date, though, we have no statistics on workflows created, workflows executed, or CyREST endpoints called. We hope to collect these in the future. However, since updating RCy3 to work with CyREST and releasing as version 2.0 in April 2018, it has risen to rank near the top 200 packages in Bioconductor, averaging ~ 800 downloads per month (up from ~ 200). py2cytoscape sees about 500 downloads/month from GitHub and the PyPI Python package index.

### External workflows enabled

The Cytoscape community has used Cytoscape Automation to create Python and R workflows that successfully load network data, profile it, perform complex layouts and styles, then return renderings.

**Fig. 5:**
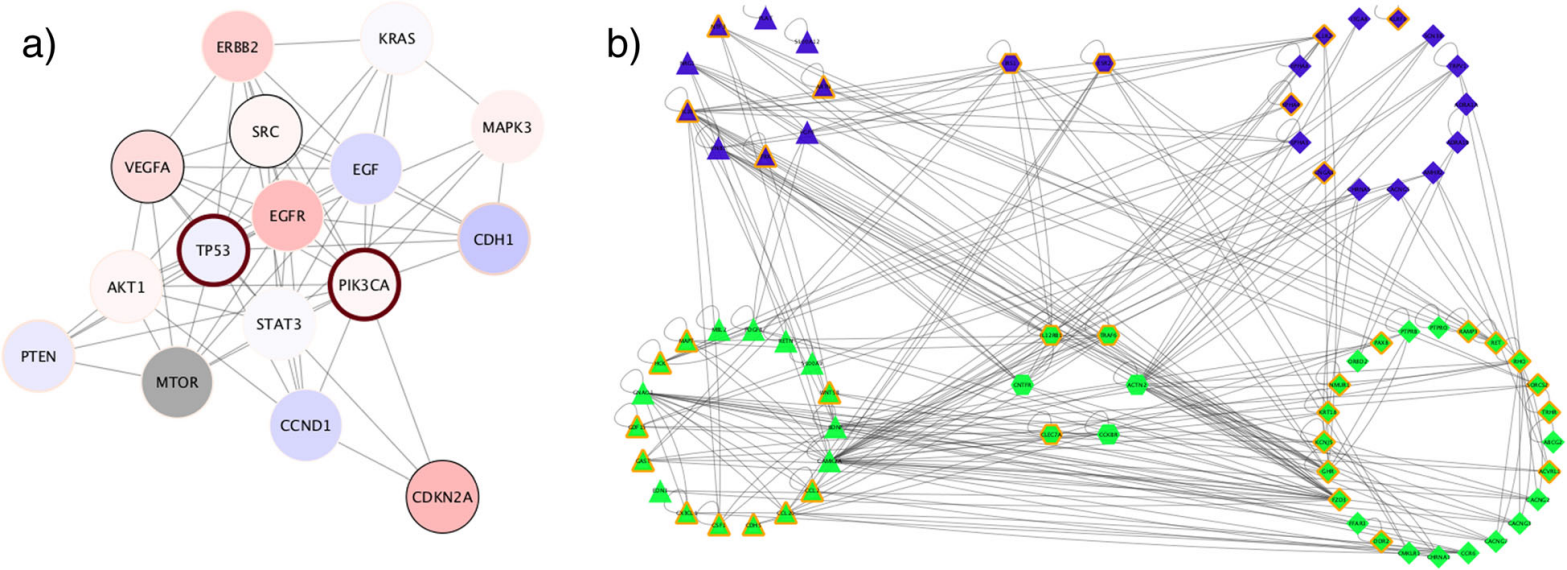
Results of Cytoscape Automation workflow execution in Python and R. **a** Uses multiple Cytoscape apps to load and analyze two data sets, then combines them to show critical genes. **b** Uses multiple R libraries and analyses to create a network that is then laid out and styled in Cytoscape

Figure 5a shows one result of the Python “advanced cancer networks and data” workflow referenced above. It loads ovarian cancer and breast cancer disease networks by calling Cytoscape’s String app [33], determines a relevant gene neighborhood by calling Cytoscape’s Diffusion app [34], and ends up with a styled and laid out subnetwork of critical breast cancer genes.

Figure 5b shows the result of the R “Isserlin_PPI_network_pipeline_R” workflow referenced above. It is a ligand-receptor network showing a number of interactions. The workflow reads ligands and receptors from Biomart by calling R libraries. Interactions are fetched from iRefIndex, Biogrid, and Pathway Commons and removes duplicate interactions. After expression analysis, it constructs a representative JSON-encoded network, sends it to Cytoscape, performs a different force-directed layout on each cell type, and creates styles to differentiate cell types, protein types, and significance.

The two workflows demonstrate that Cytoscape and its apps can be integrated with Python and R best-of-breed libraries to create novel and repeatable results. Because these workflows are defined by code, they can be audited and evolved in an orderly and predictable manner. Assuming consistent input data, correct and consistent results are attained on every run (though, not necessarily identical results if non-deterministic algorithms such as some layouts are in the workflow). Without Cytoscape Automation, attaining these qualities would have required a new Cytoscape app that would have required specialized Java coding skills and several weeks to develop.

Both workflows show how multiple Cytoscape steps can be staged in sequence to reproduce multiple repeatable results. Figure 5a is actually one of ten different images produced by the “advanced cancer networks and data” workflow, which performs over 40 different Cytoscape operations and a number of intermediate calculations. The workflow executes in under 2 min on a common workstation. If performed by hand (as would be necessary without Cytoscape Automation), the time required would have been over 20 min, and given the complexity of assessing attribute values and styling networks by hand, it is unlikely that even a skilled Cytoscape operator could have produced all images consistently. This demonstrates how Cytoscape Automation enables workflows that are practically impossible under human operation and does so in a timely manner.

Note that additional R-oriented vignettes are available at the RCy3 Bioconductor site.

Note that these workflows use languages common in data sciences, but a different class of workflows can be written in Javascript and deployed inside of web apps running inside standard browsers. For example, the NDEx website [35] uses direct Javascript-based calls to CyREST endpoints in order to enable a user to download a network from the NDEx database into a running Cytoscape instance—the transfer is initiated when the user clicks on a network page’s Cytoscape icon. From there, a user can use Cytoscape to explore, analyze, and visualize shared networks, thus sparing the NDEx authors from having to duplicate Cytoscape features in the NDEx website. In this mode, Cytoscape Automation achieves application integration not previously economical.

### Cytoscape Automation apps

In December 2017, we launched a campaign calling on all Cytoscape app writers to upgrade their existing apps to enable Automation calls, referring them to an extensive Wiki and FAQ document written to inform and enable their work. To date, Automation features have been added to 4 core apps (delivered with Cytoscape, listed in underlined italics) and 34 App Store apps:

**Table:** 

aMatReader	cyChart	eXamine	RINalyzer
AutoAnnotate	*CyNDEx-2*	GeneMANIA	RINspector
BridgeDb	Cyni Toolbox	ID Mapper	setsApp
cddApp	Cyrface	KEGGScape	stringApp
chemViz2	CyTargetLinker	MCODE	structureViz2
ClueGO	CytoCopteR	Omics Visualizer	Synapse Client
clusterMaker2	*Diffusion*	PathLinker	WikiPathways
*copycatLayout*	DisGeNET-app	PSFC	WordCloud
CyAnimator	enhancedGraphics	ReactomeFIPlugIn	yFiles
*cyBrowser*	EnrichmentMap		

While each app documents its endpoints via Swagger pages, significant discussion and examples are presented in separate publications in F1000 Research’s Cytoscape Automation app collection [36].

Note that core apps follow semantic versioning guidelines, thereby guaranteeing that updates will not break workflows supported in previous versions. To the extent that other apps follow these guidelines, they commit to the same guarantee.

## Discussion

Existing biological workflow systems (e.g., Galaxy, Taverna, GenePattern [37], bioKepler [38], and implementations of Common Workflow Language [39]) are capable of executing workflows on networks, but they do not leverage functionality available in Cytoscape and its apps. Their forte is the execution of a wide range of biological analysis tools and in a portable and scalable way across a variety of software and hardware environments. In contrast, Cytoscape Automation leverages a wide range of *network-specialized* Cytoscape and app features using a single Cytoscape instance running on a workstation, though a wide range of biological analysis tools can also be executed.

### Cytoscape Automation and workflow systems

In most workflow systems (including general programming languages such as Python, R, and Javascript and biological workflow engines such as Galaxy, Taverna, and CWL engines), workflows are constructed by calling a utility or library function, using its result in some calculation or transformation (called interstitial code), passing the result to a different utility or library function, and so on. Often, the workflow itself maintains state as variables, and the utilities and library functions are stateless—their output depends solely on their inputs. Workflows based on Cytoscape Automation are different because Cytoscape maintains network state and Cytoscape Automation functions create, query, or change networks—workflows calling Cytoscape Automation functions have state *shared* between the workflow and Cytoscape.

Cytoscape Automation functions support sequential calls in a single thread of execution, emulating operations performed by a human—the function does not return a result to its caller until the function is finished. Additionally, functions implemented entirely within the Cytoscape core are guaranteed to execute without soliciting input from a user, thereby enabling unsupervised execution. Functions implemented in Cytoscape apps *should* provide this guarantee, but that choice is left to the app author. (Note that the workflow itself remains free to solicit user or other external input as appropriate.)

While workflow systems are free to execute Cytoscape Automation workflows comprised of multiple parallel threads, Cytoscape Automation itself makes no guarantees regarding the order of function execution or termination and does not guarantee that function executions will not conflict with each other. For example, executing a network layout at the same time as updating network attributes may produce an unpredictable layout. Similarly, simultaneous calls to update network attributes may have unpredictable (and harmful!) effects on the network attributes.

As a rule of thumb, workflows should themselves serialize all operations performed on a single network. Simultaneous execution involving *different* networks will produce consistent and correct results without being serialized. Functions implemented in Cytoscape apps *should* support these rules, too, but doing so requires the author to have written them to be re-entrant (e.g., independent of global variables).

While Cytoscape Automation does not directly support checkpointing or re-execution features found in some workflow engines, it can assist a workflow author in simulating these features. The state of all networks can be saved and restored to/from a local file (using the POST and GET operations on the /v1/session endpoint) or to/from an NDEx repository (using the POST and GET /cyndex2/v1/networks endpoints). Note that restoring a network changes all Cytoscape IDs associated with collections, networks, views, nodes, edges, and every other property, thereby invalidating any IDs maintained as state by a workflow—a workflow author should take care to refresh this state via appropriate Cytoscape Automation queries after a restore operation.

Cytoscape Automation does not provide history, provenance, and other metafunctions associated with workflow execution. It relies on the workflow system to provide these features.

The following example shows how a general purpose language (e.g., Python) can be used to create a workflow that shares state with Cytoscape and use interstitial code to perform novel functions and create new Cytoscape state. Cytoscape is called to create a network of ovarian cancer genes from the STRING database, then interstitial code fetches STRING’s gene annotations from Cytoscape and creates a list of genes in the top 25th quantile of top scoring diseases. Finally, Cytoscape is called again to create a network using that gene list:

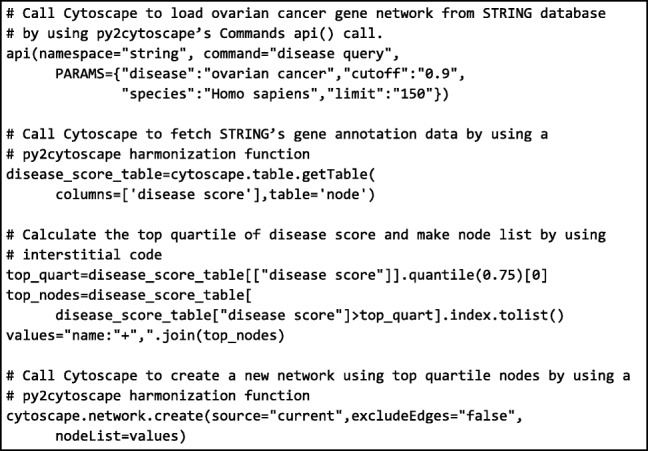



**Fig. 6:**
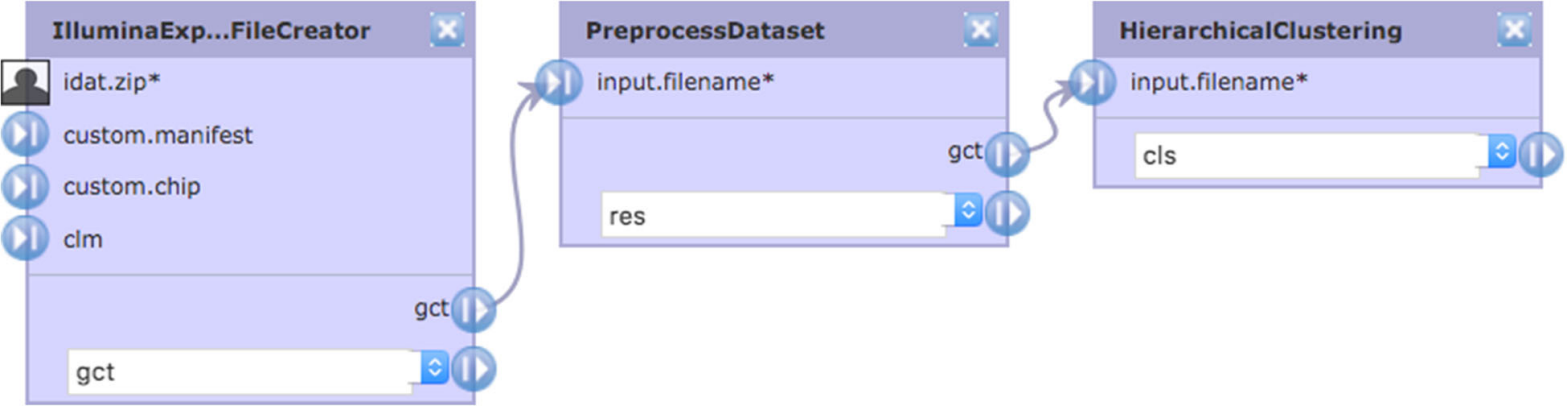
A three-step GenePattern workflow shown by the GenePattern Pipeline Editor. The Illumina Expression File Creator step creates a GCT file from a zip of Illumina IDAT files. The Preprocess Dataset step normalizes the GCT data, and the Hierarchical Clustering step performs clustering on genes. The second step was created by GenePattern staff to avoid adding parameters to the first or third steps

Systems that define a workflow as a pipeline of functional blocks linked together by data flows (e.g., GenePattern [37], as shown in Fig. 6) are challenged to maintain state or provide interstitial code such as the top quartile calculation. To the extent such systems enable the authorship of new functional blocks, Cytoscape Automation workflows can be implemented using a general purpose language and then incorporated (and reused) as a functional block.

Note that GenePattern Notebook [40] is a new Jupyter-based workflow system that can use general programming languages to orchestrate existing analyses and use their results to create complex control flows and data-dependent processing. GenePattern Notebooks can use Cytoscape Automation (including the py2cytoscape harmonization library) to create Cytoscape workflows and integrate them with other analytics.

### Workflow publishing

Biological workflow systems vary in how they envision their communities sharing workflows. GenePattern Notebook provides a web-based repository that allows users to check in a workflow and then share it with others or the public. Such notebooks can be imported directly within the GenePattern Notebook development system. Taverna enables sharing through the myExperiment [41] social network. Other systems advise that workflows be stored and shared in a version control system (VCS) such as GitHub.

Common practice in the Cytoscape Automation community is to store workflows and artifacts in GitHub and reference them from academic papers or include them as supplementary material as appropriate. This is particularly feasible because such workflows are contained in easily readable text files for which GitHub viewers (e.g., nbviewer) are available, GitHub enables sharing and versioning of workflows, GitHub has become a common tool in many biologists’ toolbelt, and GitHub can be readily searched. The 2017 CyREST Challenge produced several such examples.

A working example is a 2019 Bader Lab pathway enrichment analysis paper [42] where R-based Cytoscape Automation is delivered as supplementary material (protocol 3), and the full workflow is delivered in the GitHub repository named in its “Data availability” section. Note, too, that the paper’s “Procedure” section recites a long list of manual steps, much of which can be replaced in a less ambiguous, more reproducible way by automated workflows.

### User experience

Cytoscape Automation functionality is delivered in all Cytoscape downloads (over 17,600 per month), and in the interests of privacy, usage of individual features is not counted. Furthermore, given the typically long lag between research and paper publishing, we are only now starting to see published papers that leverage automation. Instead, we infer user interest and feedback through automation library downloads, tutorial attendance, and online posts.

Since the original Cytoscape Automation tutorial at the ISMB/Prague 2017, six more major multi-hour public tutorials have been delivered by the National Resource for Network Biology (nrnb.org). All tutorials were well and enthusiastically attended, with an average attendance of 40, and most of the class completed class exercises without difficulty.

Since January 2018, the Cytoscape help desk has tallied 68 threads relating to Cytoscape Automation, out of 710 total threads (approximately 9.5% of total worldwide support). In the same period, 76 issues were posted on the project’s GitHub, and 49 were closed (64%).

Note that the definition of the RCy3 harmonization library for automation (~ 800 downloads per month) was designed by a working group of 13 users and 5 Cytoscape core developers. The Cytoscape Automation design was responsive to the RCy3 design, and two users (e.g., Isserlin [42]) have since published research papers in which Cytoscape Automation was part of their methodology. They chose to implement Cytoscape Automation workflows because they already knew the workflow languages (Python and R) and were *already* using them and associated libraries to implement parts of their experiment protocol. Their workflows also reproduced their Cytoscape-focused steps and automated numerous tedious and error-prone steps via both Cytoscape Automation calls and interstitial code.

Finally, as further evidence of its usefulness in reproducibility, Cytoscape Automation is currently used to test automation-enabled apps (Diffusion [43] and CyNDEx-2 [44]) as well as Cytoscape itself.

## Future development

Cytoscape Automation features will continue towards providing frictionless interaction between all components of Cytoscape’s ecosystem of authors, services, and applications, particularly in the following areas:

**Table:** 

Additional apps	- Upgrade additional core and store apps to support Automation calls
App Store support	- Improve identification of Automation-supporting apps - Provide documentation of API calls in the app’s store page - Provide access to app-specific R and Python harmonization libraries
Workflow publishing	- Create a repository of workflows for use and evolution
App testing harness	- Create a workflow-based framework for testing Automation endpoints

While Cytoscape core developers will improve the infrastructure components, most value will be contributed by the Cytoscape community as it enables Automation in more apps and creates workflows that leverage Automation.

Note that the execution of Cytoscape functionality (as both core features and apps) without requiring user intervention is a milestone in the path to our long-term goal of creating the so-called headless Cytoscape, which can execute as a stand-alone service independent of a keyboard, mouse, or display.

## Summary

In this paper, we showed how Cytoscape Automation extends Cytoscape to enable reproducible, shareable, and extensible network biology workflows that can be economically built using common programming languages (e.g., Python, R, and Javascript) in common environments (e.g., Jupyter and RStudio).

The key to Cytoscape Automation is its improvements to facilities already offered in CyREST. We created standards that (1) enable Commands and Cytoscape apps to be called through CyREST and (2) encourage high-quality documentation of CyREST endpoints using state-of-the-art documentation systems (such as Swagger) and interactive call prototyping. As a result, there are now 34 Cytoscape apps that can be called via CyREST, and over 150 Cytoscape Functions and 120 Commands have been documented.

Using the specific examples in this paper and on the Cytoscape Wiki, an app author can add Cytoscape Automation to an existing app, and a bioinformatician can create novel network biologic workflows as orchestrations of Cytoscape functions, complex custom analyses, and best-of-breed external tools and language-specific libraries.

We expect that Cytoscape Automation will enable the exchange and rapid evolution of workflows that integrate Cytoscape-based network analysis and visualization. The services, software, and documentation resources that comprise the Cytoscape Automation ecosystem will play an integral role in making these workflows scalable, replicable, and of high value.

## Additional file

Additional file 1: Review history. (DOCX 13 kb)

## Data Availability

CyREST software is available as part of Cytoscape: https://cytoscape.org/download.html [45] Latest source code of cyREST: https://github.com/cytoscape/cyREST [46] Fixed source code reference for CyREST (v3.8): 10.5281/zenodo.2798856 [47] The Cytoscape Automation examples: http://automation.cytoscape.org [12] The Cytoscape Automation FAQ and Wiki: http://automation.cytoscape.org [12] The Cytoscape Automation RCy3 harmonization library source: https://github.com/cytoscape/RCy3 [48] The Cytoscape Automation RCy3 official release: https://www.bioconductor.org/packages/release/bioc/html/RCy3.html [10] The Cytoscape Automation py2cytoscape harmonization library source: https://github.com/cytoscape/py2cytoscape [9] The Cytoscape Automation Python official release: https://pypi.org/project/py2cytoscape [27] License for cyREST, py2cytoscape, and all example workflows: MIT: http://opensource.org/licenses/MIT [49]
