# Supplementary material for: Cytoscape Automation: empowering workflow-based network analysis
Source: Genome Biol. 2019 Sep 2;20:185. doi: 10.1186/s13059-019-1758-4 (PMC6717989; doi:10.1186/s13059-019-1758-4)
Supplement: Supplementary file 1 — Review history. (DOCX 13 kb) [file 13059_2019_1758_MOESM1_ESM.docx]

Review History

**First round of review**

**Reviewer 1**

**Were you able to assess all statistics in the manuscript, including the appropriateness of statistical tests used?**

There are no statistics in the manuscript.

**Were you able to directly test the software?**

No

**Comments to author:**

This paper describes an interesting set of software tools for the automation of processes within Cytoscape.

Cytoscape is an impressive and widely used application that has had large impact on network biology. These developments add new functionality and will be potentially interesting to many. However, the current presentation of this work does not allow a full evaluation of its utility.

Please consider the following questions:

-Is the Software likely to be of broad utility? Is it easy to install and use?

Cytoscape is widely used, so this software does potentially have broad utility. The authors provide several examples of where this software has been used, but there are no details of any usability assessment or details of any user experiences.

-Does the software represent a significant advance over previously published tools (as demonstrated by direct comparison with available related software)?

The software enables automated workflows to be constructed from Cytoscape resources, so I would expect a comparison to existing workflow systems. There are a few mentioned, but there is no comparison. There are a number of example workflows provided, but they are provided without much detail of their functionality or evaluation of their performance. I would expect a deeper discussion about what the workflow versions provide that were not available already (e.g. in terms of scientific reproducibility, speeding up analyses, remote hosting for service provision etc). At the very least - do the workflow versions give the same results?

-Are the methods appropriate to the aims of the study, are they well described, and are necessary controls included?

The aims of the study are not well articulated, although the software provides a general solution and the paper is presented as such.

-Are the conclusions adequately supported by the data shown?

As there is no evaluation of the software, it is difficult to say whether the software is fit for purpose.

The software works with a previous version of cytoscape and this is a point of concern. The functionality of this software relies on app developers providing REST interfaces to their work. App developers will be less motivated to provide extra functionality for older versions of cytoscape. What is required to bring this software up to date? Publishing something that is already out of date is not an ideal situation for the journal or for the authors. Can the authors sustain the software and keep it up to date with the cytoscape?

**Reviewer 2**

**Were you able to assess all statistics in the manuscript, including the appropriateness of statistical tests used?**

There are no statistics in the manuscript.

**Were you able to directly test the software?**

Yes

**Comments to author:**

The manuscript by Otasek et al presents Cytoscape Automation, updated scripting capabilities for Cytoscape. Cytoscape and the associated apps present a fairly unique toolset for bioinformatics and beyond, with a strong user community; the capacity to script Cytoscape is an often required feature. This manuscript represents a significant, though incremental step in the development of Cytoscape's scriptability. While it was already possible to script Cytoscape, interfaces and documentation have been harmonised and improved, the scriptability of external apps has been added, and a significant set of apps implementing this capability is presented. However, as the authors discuss themselves, there is likely much more work, and likely interface changes coming on the way to a "headless" Cytoscape.

The presentation of workflows themselves is a weak point in the manuscript. The authors only briefly comment "In the future, we hope to provide a standard way for workflow authors to create and disseminate workflows they create.". A more thorough discussion of at least the vision of the authors in this area, also in the context of efforts like CDL, would be desirable. Ref 25 points to "number of sample workflows", but two out of four "Teaser" links (Mark Grimes, Ruth Isserlin) result in 404 errors (accessed 14/4/2019).

I am surprised that the set of F1000 publications on Cytoscape automation (PMID:29983926 and related) is not referenced.

**Authors Response**

**Point-by-point responses to the reviewers’ comments:**

Reviewer 1:

This paper describes an interesting set of software tools for the automation of processes within Cytoscape.

Cytoscape is an impressive and widely used application that has had large impact on network biology. These developments add new functionality and will be potentially interesting to many. However, the current presentation of this work does not allow a full evaluation of its utility.

Please consider the following questions:

Is the Software likely to be of broad utility? Is it easy to install and use?

Cytoscape is widely used, so this software does potentially have broad utility. The authors provide several examples of where this software has been used, but there are no details of any usability assessment or details of any user experiences.

*•	Thank you. As evidence of its broad use, we added download, installation and usage statistics to the Introduction section (page 1) and the Results section (page 18). We also added a new User Experience section page 23) that explains how we have evaluated Cytoscape Automation usability and user experiences, given its newness.*

*•	As evidence of ease of use, we added significant discussion in the Introduction (page 1), particularly focusing on familiarity of the user base with languages, environments and technology; use of widely used Swagger documentation, and the significant documentation and examples provided in the Cytoscape Automation Wiki. We also updated download and use statistics throughout, as they show that Cytoscape’s popularity (and specifically Cytoscape Automation components) continues to grow.*

*•	As clarification of the ease of use for CyREST call prototyping, we have added emphasis in the “New Interactive CyREST call protoyping” section (page 10) that interactive prototyping doesn’t require any programming skills at all.*

Does the software represent a significant advance over previously published tools (as demonstrated by direct comparison with available related software)?

The software enables automated workflows to be constructed from Cytoscape resources, so I would expect a comparison to existing workflow systems. There are a few mentioned, but there is no comparison. There are a number of example workflows provided, but they are provided without much detail of their functionality or evaluation of their performance. I would expect a deeper discussion about what the workflow versions provide that were not available already (e.g. in terms of scientific reproducibility, speeding up analyses, remote hosting for service provision etc). At the very least - do the workflow versions give the same results?

*•	These are excellent points. To address them, we added a new Discussion section (Workflow Language Capabilities on page 21), which identifies a number of relevant workflow systems and compares them to Cytoscape Automation. Since Cytoscape Automation’s first purpose is to execute Cytoscape steps (which differs from other workflow systems and makes Cytoscape Automation unique), it isn’t possible to compare it head-to-head to other workflow systems. However, we do explain the principles that make Cytoscape Automation superior for its purpose, including giving an annotated code example.*

*•	We updated the workflow examples in the Calling Endpoints section (page 14) to contain annotations describing each step.*

*•	We added a very relevant performance discussion in the External Workflows Enabled section (page 18), comparing the execution speedups and reproducibility of a manual workflow to Cytoscape Automation’s program-driven workflows, which is the use case most important to the Cytoscape Automation feature. We also addressed reproducibility under non-deterministic algorithms. In the User Experience section (page 23), we point out that because these workflows consistently reproduce results, they have been used in published papers and they are being used to test apps and Cytoscape itself.*

Are the methods appropriate to the aims of the study, are they well described, and are necessary controls included?

The aims of the study are not well articulated, although the software provides a general solution and the paper is presented as such.

*•	Thank you for bringing this up. By “aims of the study”, we intend that Cytoscape Automation address the multiple issues identified in the first paragraph of the Introduction section (page 1). We improved the remainder of the Introduction section to explain that we are addressing these deficiencies, and give an overview of how we’re addressing them.*

Are the conclusions adequately supported by the data shown?

As there is no evaluation of the software, it is difficult to say whether the software is fit for purpose.

The software works with a previous version of cytoscape and this is a point of concern. The functionality of this software relies on app developers providing REST interfaces to their work. App developers will be less motivated to provide extra functionality for older versions of cytoscape. What is required to bring this software up to date? Publishing something that is already out of date is not an ideal situation for the journal or for the authors. Can the authors sustain the software and keep it up to date with the cytoscape?

•	*Thank you for raising this point. We understand that the reviewer is concerned with which Cytoscape versions Cytoscape Automation requires, and how to guarantee that workflows written for one version of Cytoscape (and apps) will continue to work as Cytoscape (and apps) evolve. To make this clear, we added an explanation to the end of the Introduction section (page 1) stating that Cytoscape v3.7 is required, that Cytoscape’s commitment to semantic versioning principles is re-asserted, that CyREST is maintained as part of Cytoscape (and therefore adheres to semantic versioning), and that independent app authors are (and have been) encouraged to adhere to semantic versioning, too. This is now restated, too, in the Cytoscape Automation Apps section (page 20).*

*•	There is a short paragraph at the end of the “New CyREST access to Cytoscape…” section (page 5) that explains a Command deprecation of sorts, though not to the degree of desupporting old Command style usage.*

Reviewer 2:

The manuscript by Otasek et al presents Cytoscape Automation, updated scripting capabilities for Cytoscape. Cytoscape and the associated apps present a fairly unique toolset for bioinformatics and beyond, with a strong user community; the capacity to script Cytoscape is an often required feature. This manuscript represents a significant, though incremental step in the development of Cytoscape's scriptability. While it was already possible to script Cytoscape, interfaces and documentation have been harmonised and improved, the scriptability of external apps has been added, and a significant set of apps implementing this capability is presented. However, as the authors discuss themselves, there is likely much more work, and likely interface changes coming on the way to a "headless" Cytoscape.

The presentation of workflows themselves is a weak point in the manuscript. The authors only briefly comment "In the future, we hope to provide a standard way for workflow authors to create and disseminate workflows they create.". A more thorough discussion of at least the vision of the authors in this area

*•	Thank you for these excellent points. We addressed the workflow dissemination question in the Workflow Publishing subsection of the new Discussion section (page 21), where we argue that GitHub has become a commonly used repository for sharing, versioning and discovery, and we gave an example of how a published paper is making use of it.*

, also in the context of efforts like CDL, would be desirable.

*•	This is an excellent point. While we understand that the reviewer identified “CDL”, we searched for CDL and determined that “CWL” was most likely the intended reference. To address this, we added the Discussion section (including the Workflow Language Capabilities sub-section, page 21) to compare and contrast Cytoscape Automation and existing workflow systems (i.e., CWL and others).*

Ref 25 points to "number of sample workflows", but two out of four "Teaser" links (Mark Grimes, Ruth Isserlin) result in 404 errors (accessed 14/4/2019).

•	*Thank you for noticing this. The GitHub repository was reorganized after this paper was drafted. We have repaired the links in the Workflow Examples section (page 17) and have organized the repository to be less susceptible to link breakage. Also, we updated the workflow names in the External Workflows Enabled section (page 18) to match.*

I am surprised that the set of F1000 publications on Cytoscape automation (PMID:29983926 and related) is not referenced.

•	*Thank you for noticing this … we were surprised, too. We have added a reference to the entire collection, and added it to the Cytoscape Automation Apps section (page 20). Note that we also updated that section to include the newest list of automation-enabled apps in the app store … there is a bunch more.*

**Second round of review**

**Reviewer 1**

The authors have addressed each of my comments and the new version of the manuscript gives a much clearer description of the work

The new workflows comparison section, however, is still not very clear. I am not sure I follow the arguments presented. The authors state that “a mismatch causes the addition of purpose-written connector steps, the addition of additional parameters to existing

steps, or both. While such steps can be reused in subsequent workflows, the time and funding cost of creating new steps or modifying existing steps retards the flexibility and liveliness of workflow development, while increasing platform maintenance and training costs. In contrast, Cytoscape Automation workflows are implemented using general purpose

programming languages, which enable passing state as either function results or direct queries to Cytoscape. A connector step would be written as discrete interstitial code or a call to packaged routines in libraries”.

It is difficult to see how this is different from many other workflow systems and perhaps should not be presented as a unique feature. Many workflow systems allow simple scripting in general purpose programming languages. It seems unlikely that this activity would be a much higher development cost than in Cytoscape Automation.

For this section, I was expecting a more traditional workflow comparison – what kinds of execution are supported? Are steps executed as a linear set of operations? Can the workflow branch into multiple parallel processes? Can the user make decisions at run-time? Does it allow retries or partial reruns? When the workflow is run, does it keep a history of the run with its parameters, inputs and intermediate inputs/outputs. I basically wanted to know how much of a workflow system it is.

The purpose of Cytoscape Automation is not to reinvent workflows, but to allow cytoscape tools to be linked together. It is not essential for there to be a unique feature that other workflow systems lack, but understanding how similar it is to others (and whether it can be exported in the CWL) will help to improve the functionality in the long run.

**Reviewer 2**

In this updated manuscript, Otasek et al have comprehensively addressed the points I raised.

**Authors Response**

**Point-by-point responses to the reviewers’ comments:**

The authors have addressed each of my comments and the new version of the manuscript gives a much clearer description of the work The new workflows comparison section, however, is still not very clear. I am not sure I follow the arguments presented. The authors state that “a mismatch causes the addition of purpose-written connector steps, the addition of additional parameters to existing steps, or both. While such steps can be reused in subsequent workflows, the time and funding cost of creating new steps or modifying existing steps retards the flexibility and liveliness of workflow development, while increasing platform maintenance and training costs. In contrast, Cytoscape Automation workflows are implemented using general purpose programming languages, which enable passing state as either function results or direct queries to Cytoscape. A connector step would be written as discrete interstitial code or a call to packaged routines in libraries”. It is difficult to see how this is different from many other workflow systems and perhaps should not be presented as a unique feature. Many workflow systems allow simple scripting in general purpose programming languages. It seems unlikely that this activity would be a much higher development cost than in Cytoscape Automation. For this section, I was expecting a more traditional workflow comparison – what kinds of execution are supported? Are steps executed as a linear set of operations? Can the workflow branch into multiple parallel processes? Can the user make decisions at run-time? Does it allow retries or partial reruns? When the workflow is run, does it keep a history of the run with its parameters, inputs and intermediate inputs/outputs. I basically wanted to know how much of a workflow system it is.

*• Thank you for these excellent questions. They brought out a substantial omission that we’re happy to fix. To answer the questions, we reorganized and augmented the section called Workflow Language Capabilities, and renamed it Cytoscape Automation and Workflow Systems. In it, we gave background as to the structure of Cytoscape Automation workflows, and explained the capabilities of its model as compared to major workflow systems (e.g., the consequences of the Cytoscape Automation functions maintaining a stateful network model). We gave specific advice regarding workarounds to address missing features (e.g., parallel execution and checkpoints). Technically speaking, we could have written a book on these topics alone, but believe this new discussion hits the important points sufficiently to steer an interested reader.*

The purpose of Cytoscape Automation is not to reinvent workflows, but to allow cytoscape tools to be linked together. It is not essential for there to be a unique feature that other workflow systems lack, but understanding how similar it is to others (and whether it can be exported in the CWL) will help to improve the functionality in the long run.
